# Biological effect of bone marrow mesenchymal stem cell- derived extracellular vesicles on the structure of alveolar bone in rats with glucocorticoid-induced osteoporosis

**DOI:** 10.1186/s12891-023-06276-2

**Published:** 2023-03-17

**Authors:** Aya S. Sedik, Khadiga Y. Kawana, Azza S. Koura, Radwa A. Mehanna

**Affiliations:** 1 Department of Oral Biology, Faculty of Dentistry, Alexandria University, Alexandria, Egypt; 2 Department of Physiology, Faculty of Medicine, Alexandria University, Alexandria, Egypt; 3 Center of Excellence for Research in Regenerative Medicine and Applications (CERRMA), Faculty of Medicine, Alexandria University, Alexandria, Egypt

**Keywords:** Glucocorticoids, Osteoporosis, Mesenchymal stem cells, Extracellular vesicles

## Abstract

**Background:**

Glucocorticoids are used for the treatment of autoimmune disorders; however, they can elicit several side effects such as osteoporosis. Several approaches can be made to treat glucocorticoid-induced osteoporosis, including the use of stem cells. However, the therapeutic effect of mesenchymal stem cells depends on its released factors, including extracellular vesicles. Extracellular vesicles have been recognized as important mediators of intercellular communication as they participate in many physiological processes. The present study was designed to investigate the effect of bone marrow mesenchymal stem cells derived extracellular vesicles on the structure of alveolar bone in rats with glucocorticoid-induced osteoporosis.

**Methods:**

Thirty adult albino male rats were divided into 3 groups: control group (CG), glucocorticoid-induced osteoporosis (GOG) and extracellular vesicles treated group (ExTG). Rats in the GOG and ExTG groups were injected with methylprednisolone acetate (40 mg/kg) intramuscularly in the quadriceps muscle 3 times per week for three weeks in the early morning. Afterwards, the rats in GOG group received a single vehicle injection (PBS) while each rat in the ExTG group received a single injection of extracellular vesicles (400 μg/kg suspended in 0.2 ml PBS) in the tail vein. Rats were euthanized 1 month after injection. Mandibles were dissected and the molar segments were prepared for histological preparation, scanning electron microscopy (SEM), and energy dispersive x-ray (EDX).

**Results:**

Histology and scanning electron microscopyof bone tissue showed alveolar bone loss and bone resorption in the GOG group. while in the ExTG group, alveolar bone demostrated normal bone architecture. EDX showed that calcium percentage in GOG group was lower than ExTG group,which showed no statistically significant difference from the control group.

**Conclusions:**

Extracellular vesicles may be a promising treatment modality in the treatment of bone diseases and in bone regeneration. However, further research is needed before stating that extracellular vesicles s can be used to treat bone disorders especially when translating to humans.

## Highlight

• Extracellular vesicles derived from bone marrow mesenchymal stem cells can promote alveolar bone regeneration evident by restoration of the normal architecture and mineral balance in the alveolar bone after the glucocorticoid’s induction of osteoporosis.

## Background

Glucocorticoids (GCs) are a group of drugs that have multiple applications clinically because of their immunosuppressive and anti-inflammatory properties [1]. In addition, GCs regulate many cellular functions including homeostasis, metabolism, cognition, and inflammation [2].

GCs perform their functions by two mechanisms either genomic or non-genomic pathway [3]. In the genomic pathway, Glucocorticoids perform pharmacological and physiological functions by binding to GR; the glucocorticoid receptor [4]. Following GC-GR binding, conformational change occurs in the receptor, allowing this complex to translocate to nucleus, where it binds to chromatin and, as a result, affects target gene expression [3].

Moreover, chromatin binding to GR is accomplished by AP-1 (activation protein-1) transcription factor so the receptor can recognize target sequences [5]. Homodimerization of the receptor occurs and thus binds to GREs (Glucocorticoids response elements) [6].

Furthermore, “GREs” are found in the target gene promoter region, and when the GC-GR binds to GCs-response elements, it causes the transcription of interleukin 10 (IL-10) anti-inflammatory cytokines. This process is known as transactivation, and it is related to the adverse effects developed by GCs [7].

Furthermore, GCs use trans-repression as another mode of action. It demonstrates the GC–GR complex ability to inhibit the trans-activation of certain transcription factors, as nuclear factor kappa B (NFκB) and AP-1, through indirect or direct interactions [8]. Trans-repression inhibits pro-inflammatory mediators such as (interleukin IL1, IL2, IL6, IL8, tumour necrosis factor (TNF), interferon gamma) and prostaglandins involved in the inflammatory response, and it has become one of the most important explanations for the immune-suppressive and anti-inflammatory effects of GCs [9].

However, the non-genomic pathway is faster and is mediated by interactions between the membrane-bound GR or intracellular GR. A cascade of effects is initiated within a limited time after receptor activation that includes impairing the release of arachidonic acid, the inhibition of phospholipase A2, which produces inflammatory cytokines, and regulation of apoptosis in thymocytes [10].

At pharmacological dose, GCs affect intestinal and renal calcium level as well as growth and sex hormone. For instance, glucocorticoids reduce calcium absorption in the duodenum and inhibit calcium renal reabsorption which affect calcium homeostasis negatively; and thereby, affect bone metabolism [11]. Furthermore, excess glucocorticoids cause premenopausal symptoms in women and hypogonadism in men, which have a negative impact on bone health [12].

Since transactivation accounts for most of the adverse effects related to glucocorticoids, thereby, many attempts were made to develop drugs with GCs-like action (e.g., immune suppression); however, limited success was achieved [13], however, pharmacological treatment usually targets the symptoms and complications. In addition, pharmacotherapy can take a long period of time to be effective and can elicit undesirable side effect. Moreover, long term hormone replacement therapy can increase the risk of cerebral infarction, breast cancer, and stroke [14].

Therefore, regenerative medicine brings new hope to a lot of patients with skeletal disorders due to the differentiation and self-renewal ability of the stem cells [15].

Stem cell therapy is an advanced and promising scientific field [16]. The concept of stem cell therapy is dependent on the human body potential to regenerate. Stem cells are regarded as excellent candidates for replacement therapies, gene therapy, and cancer therapeutics in regenerative medicine and tissue engineering. Also, they are used to fight major diseases as connective tissue or bone disorders, heart disease, neural defects, and hematological disorders [17].

Mesenchymal stem cells (MSCs) are considered as multipotent stem cells that differentiate into chondrocytes, osteoblasts, adipocytes, and muscle cells [18]. Furthermore, mesenchymal stem cells express genes involved in cell-to-cell communication as well as extracellular matrix proteins like interstitial type I collagen, type IV collagen, fibronectin, and laminin. They also secrete cytokines like interleukin (IL)-8, IL-7, IL-11, stem cell factor (SCF), and stromal-derived-factor-1 (SDF-1) that regulate hematopoietic stem cell homing into the BM by renewing the stromal microenvironment required for hematopoiesis [19]. Bone marrow-mesenchymal stem cells (BMMSCs) are easily isolated, proliferative andmultipotent stem cells. BM-MSCs differentiate into osteoblasts, osteocytes, and chondrocytes and thus has significance in bone regeneration [20]. They release a variety of osteogenic-related factors under controlled conditions. They also show promising results in bone repair and healing as well as soft tissue injuries in vivo [21].

Furthermore, BM-MSCs has osteogenic effect that is mediated systemically by hormones like estrogens, GCs, and PTH and locally by GFs as TGF-β, bone morphogenetic protein (BMPs) and FGF-2 (fibroblast growth factor-2). These factors initiate several intra-cellular signaling pathways, resulting in the expression of several transcription factors in MSCs and, consequently, the differentiation of osteoblasts rather than chondrocytes or adipocytes [22].

Nevertheless, the utilization of MSCs in bone regeneration was postulated by their ability to differentiate into osteoblasts and their ability to replace damaged tissue; however, it was assumed that mesenchymal stem cells secrete certain factors that regulate many cellular processes needed for bone regeneration as angiogenesis and osteogenesis [23, 24].

Although MSCs are used extensively in therapy, they are expensive and present many obstacles as monitoring during processing, delicate handling, and optimum storage techniques to ensure the potency and viability of the transplanted cells [25].

Moreover, utilizing stem cells in regenerative medicine possesses crucial limitations as low survival rate of cells post-transplantation, post-administration immunological responses and reduced cell differentiation. Also, increased apoptosis following the transplantation is another major concern as apoptosis can elicit immune responses which may aggravate the disease or lead to rejection of the transplanted cells [26].

Accordingly, it was found that stem cells regeneration capacity is mediated by their paracrine action instead of their implantation and differentiation at the restoration sites [27]. Consequently, extracellular vesicles have been identified as major mediators of these paracrine actions and have been of increased interest in regenerative medicine [28, 29].

Extracellular vesicles are nano, bilipid membrane vesicles that have a variable size ranging from 30 to 150 nm [30].

Extracellular vesicles are secreted from all body cells and thus can be found in breast milk, plasma, amniotic fluid, blood, cerebrospinal fluid, saliva, urine, and synovial fluid. They carry the cargo of the parent cell and act as cell communicators [31, 32].

Intraluminal vesicles (ILVs) are extracellular vesicles precursors; they develop from the endocytic cisternae membrane and originate from inward budding of micro-domain. When the intraluminal vesicles accumulate, the cisternae transform into multivesicular bodies (MVBs). Some multivesicular bodies merge with the plasma membrane and hence, are released to the extracellular space after persisting in the cytosol; these ILVs are now known as exosomes [28, 33, 34].

Moreover, extracellular vesicles membranes are extensively studied, and they contain a broad range of lipid-anchored membrane proteins, transmembrane proteins, peripherally associated membrane proteins and soluble proteins of the extracellular vesicle’s lumen. However, the content of EVs depends on its origin [35].

Furthermore, it was found that extracellular vesicles have a significant role in invitro and in vivo bone regeneration. They control and direct MSC differentiation into the osteogenic lineage. Extracellular vesicles-derived from MSCs can be used for inducing osteogenic linage in naive stem cells [36].

However, the literature lacks enough evidence on the effect of extracellular vesicles on osteoporosis and the regenerative capacity of EVs on bone regeneration. The null hypothesis of this study stated that there is difference between control, calcium deficiency and exosomes treatment group.

Therefore, the present study investigates the biological effect of bone marrow mesenchymal stem cell-derived extracellular vesicles on the structure of alveolar bone in rats with glucocorticoid-induced osteoporosis.

## Methods

Thirty adult male Sprague Dawley albino rats six months old (200–250 g in weight) were used in this study. Animals were obtained from the animal house of Medical Research Institute, Alexandria University. They were kept under the same environmental conditions in the experimental animal house [37]. The rats were housed in specially designed wire mesh bottom cages and received water and diet throughout the experimental period, wherethe diet was the same throughout the whole experimental period. The animals were maintained under controlled cycle of temperature and humidity. The study is reported in accordance with ARRIVE guidelines". All experimental procedures were performed in accordance with the guidelines of the research ethics code approved by the Research Ethics Committee of Alexandria Faculty of Dentistry (IORG008839, 0186–11/2020) and Alexandria Faculty of Medicine who is a member in the International Council of Laboratory Animal Science organization (ICLAS-http://iclas.org/). Rats were randomly divided into three groups: 10 rats in each group including dropouts. Randomization was done by using permuted block technique [38]. The groups included a Control group (CG), glucocorticoid-induced osteoporosis group (GOG),extracellular vesicles treated group (ExTG) which are detailed below.

Before beginning the experiment, an optimizing group was assigned to evaluate the effect of the dose and duration of methyl prednisolone acetate used in this experiment on the structure of alveolar bone. Therefore, 5 rats were injected intramuscularly in the quadriceps with methyl prednisolone acetate 40 mg/kg for 3 times per week for 3 weeks in the early morning [39], then rats were euthanized and the molar segments in the alveolar bone was evaluated by scanning electron microscopy which showed rough, irregular outline of the alveolar bone and destruction in the buccal cortical plate which indicated that this dose and duration of GCs can cause osteoporotic changes in the alveolar bone.

After confirming the effect of the dose used and the duration of the drug, rats in the study groups (GOG and ExTG) were injected by methylprednisolone acetate (40 mg/kg) intramuscularly in the quadriceps muscle for three times per week for 3 weeks in the early morning. Afterwards, Rats in GOG group received a single vehicle injection (PBS) while each rat in ExTG group received a single injection of extracellular vesicles (400 μg/kg suspended in 0.2 ml PBS) [40, 41] in the tail vein [39]. Rats were euthanized by cervical dislocation method 1 month after injection. Mandibles were dissected and the molar segments were prepared for histological preparation, scanning electron microscope and energy dispersive x-ray microanalysis (EDX).

### Isolation and culture of bone marrow mesenchymal stem cells [42]

Bone marrow cells were obtained from the femur and tibia of Sprague Dawley albino rat weighing about 35-40 g. Rats’ femurs and tibias were dissected aseptically, and their ends were trimmed to expose the interior marrow shaft. The bone marrow was flushed with complete media, then re-suspended and passed through a 70-mm filter mesh in a new falcon tube to remove any bone spicules or fibers. The cell suspension was centrifuged at 1200 RPM for 5 min then resuspended in complete media [DMEM Minimum Essential Medium L.G supplemented with 10% fetal bovine serum (FBS), 100 IU ml 1 penicillin and 100 mg ml 1 streptomycin] then seeded in T-25 flask and cultured in humidified 5% CO2 incubator at 37̊ C. When adherent cells reached 80% confluence, they were split with 0.25% trypsin/ethylene diamine tetra-acetic acid (EDTA) solution in a ratio of 1:3. MSCs at passage three (P3) were used for the current experiments.

### Characterization of BM-MSCs

#### Fluorescence-activated cell sorting (FACS) [40]

At passage 3 mesenchymal stem cells were characterized by using fluorescence-labelled mono-clonal antibodies (mAb) for surface markers: CD90, CD45, CD44, CD11b, CD73 and CD 105 surface markers. Adherent cells were trypsinized, washed with phosphate buffer solution and incubated 30 min in the dark at room temperature; with mono-clonal phyco-erythrin (PE)-conjugated anti-bodies for CD44, CD105, CD73, CD11b (Abcam, Cambridge, UK), mono-clonal Allophycocyanin conjugated antibody for CD90 and FITC-conjugated antibody for CD45 (Abcam, Cambridge, UK). The cells were washed three times with phosphate buffer solution, before being re-suspended in 500 µl FACS buffer. Immuno-fluorescence was performed on viable cells using BD FACS caliber flow cytometer equipped with Cell Quest software (Becton Dickinson, New Jersey, USA).

#### Colony-forming unit assay [43]

The colony-forming potential (CFU) of the cell in culture was determined at (P3). One-hundred cells were platted in a six-well plate and incubated for fourteen days in culture media. After 14 days, the cells were washed with phosphate buffer solution, fixed, and stained for 5 min in each well with Crystal Violet (Sigma-Aldrich, USA) at 3 percent (w/v) in methanol for 5 min. at room temperature. After removing the stain, the cells were washed in distilled water. Counting the number of colonies in each well yielded the colony-formation potential, where plating efficiency was calculated as (number of colonies formed/number of cells plated) × 100. Visible colonies were counted and the number of colonies with five or more cells was estimated using a phase-contrast inverted microscope. A CFU potential of more than 40% was thought to be optimal for MSC culture.

### Preparation of extracellular vesicles derived from bone marrow mesenchymal stem cells [42]

Cells at P3 were cultured in serum-free media for 48 h, after which the conditioned media was collected and used for exosomes isolation. Conditioned serum-free media was centrifuged at 300 × g at room temperature for 5 min to remove any cell debris. The supernatant was further centrifuged at high-speed centrifugation 3000 × g at room temperature for 40 min followed by filtration through a 0.2um filter to remove large micro- vesicles. For isolating extracellular vesicles (50– 150 nm), ultracentrifugation at 120000 × g 4 C for 70 min was done.

#### EVs quantification using protein assay [44]

Extracellular vesicles were quantified by approximating the protein content in the isolated sample by BCA protein essay kit (Sigma-Aldrich, St. Louis, MO, USA). Extracellular vesicles were quantified by approximating the protein content in the isolated sample by colorimetric BCA protein essay kit following the manufactures protocol (Sigma-Aldrich, St. Louis, MO, USA) [44].

#### Transmission electron microscopic analysis of EVs [45]

A TEM was used to examine extracellular vesicles. The pellet of extracellular vesicles was dissolved in phosphate buffer solution, loaded onto copper grids, and stained with 1 percent (w/v) phospho-tungstic acid (PTA). TEM with a 120 kV accelerating voltage was used to examine the samples (JEM-1400 series 120 kV Transmission Electron Microscope, USA).

#### Zeta-sizer (Malvern, UK) [40]

To prevent aggregate formation, a diluted sample of extracellular vesicles was sonicated before being placed in the zeta sizer sample tube to determine the size of the nanoparticles. The data was analyzed, and the polydispersity index (PDI) was calculated using Malvern Panalytical Software from the United Kingdom.

#### Fluorescence-activated cell sorting (FACS)

BM-MSCs isolated extracellular vesicles were characterized by their surface tetraspanin proteins using a modified protocol [46]. Conjugated antibodies against CD9, CD63, and CD81 were used to label the extracellular vesicles. 50 μL of extracellular vesicles MicroBeads was added to the extracellular vesicles sample, vortexed and incubated for 1 h at room temperature. Following incubation extracellular vesicles bound beads were further washed in PBS/1% BSA, blocked with 10% BSA, and stained with Anti-CD9 [CD9 (Santa Cruz Biotechnology, (C-4):sc-13118) conjugated with AlexaFlour@488, Anti-CD63 Antibody (Santa Cruz Biotechnology, MX-49.129.5: sc-5275) conjugated with AlexaFlour@647, and Anti-CD81 Antibody (Santa Cruz Biotechnology, (1.3.3.22): sc-7637] conjugated with AlexaFlour@546, all in concentration 1 µg/mL. The stained sample was incubated for 1 h at room temperature then washed and resuspended in FACS buffer for further analysis using BD FACS Calibur. While negative marker used for characterizing the EVs was done by using a mitoTracker (life technologies cat# M7512,USA) which is a red-fluorescent dye that stains mitochondria.

### Methods of specimens’ examination

#### Light microscope examination

Specimens (the left molar segments) were fixed in 10% neutral-buffered formalin, washed, decalcified with 8% trichloroacetic acid, dehydrated with ascending concentrations of ethanol, cleared with xylene, and embedded in paraffin wax blocks. Sections were cut at a thickness of 4 μm and stained with Hematoxylin and Eosin (H&E) to be examined by light microscope for histological evaluation.

#### Scanning Electron Microscope (SEM) [47]

Specimens (the right molar segments) were examined by scanning electron microscope at the Electron microscope unit in the Faculty of Science Alexandria University to study the surface characterization of alveolar bone in different groups. The samples were mounted using silver paint on the specimen holder then coated to be ready for scanning electron microscopic examination.

#### Energy Dispersive X-ray (EDX) [48]

The samples were thoroughly washed with tap water, de-hydrated, and air-dried. The surfaces of the mandible of the study and control groups were exposed to x-rays using an EDX to determine the percentages of phosphorus and calcium.

#### Statistical analysis

The EDX microanalysis results were collected and analyzed. Data were fed to the computer and analyzed using IBM SPSS software package version 20.0*.* (Armonk, NY: IBM Corp**)** Qualitative data were described using number and percent. The Kolmogorov–Smirnov test was used to verify the normality of distribution Quantitative data were described using range (minimum and maximum), mean, standard deviation, median and interquartile range (IQR). Significance of the obtained results was judged at the 5% level. The used tests were F-test (ANOVA) for normally distributed quantitative variables, to compare between more than two groups, and Post Hoc test (Tukey) for pairwise comparisons.

## Results

### Stem cell characterization

#### Morphological characterization of BM-MSCs

**Fig. 1:**
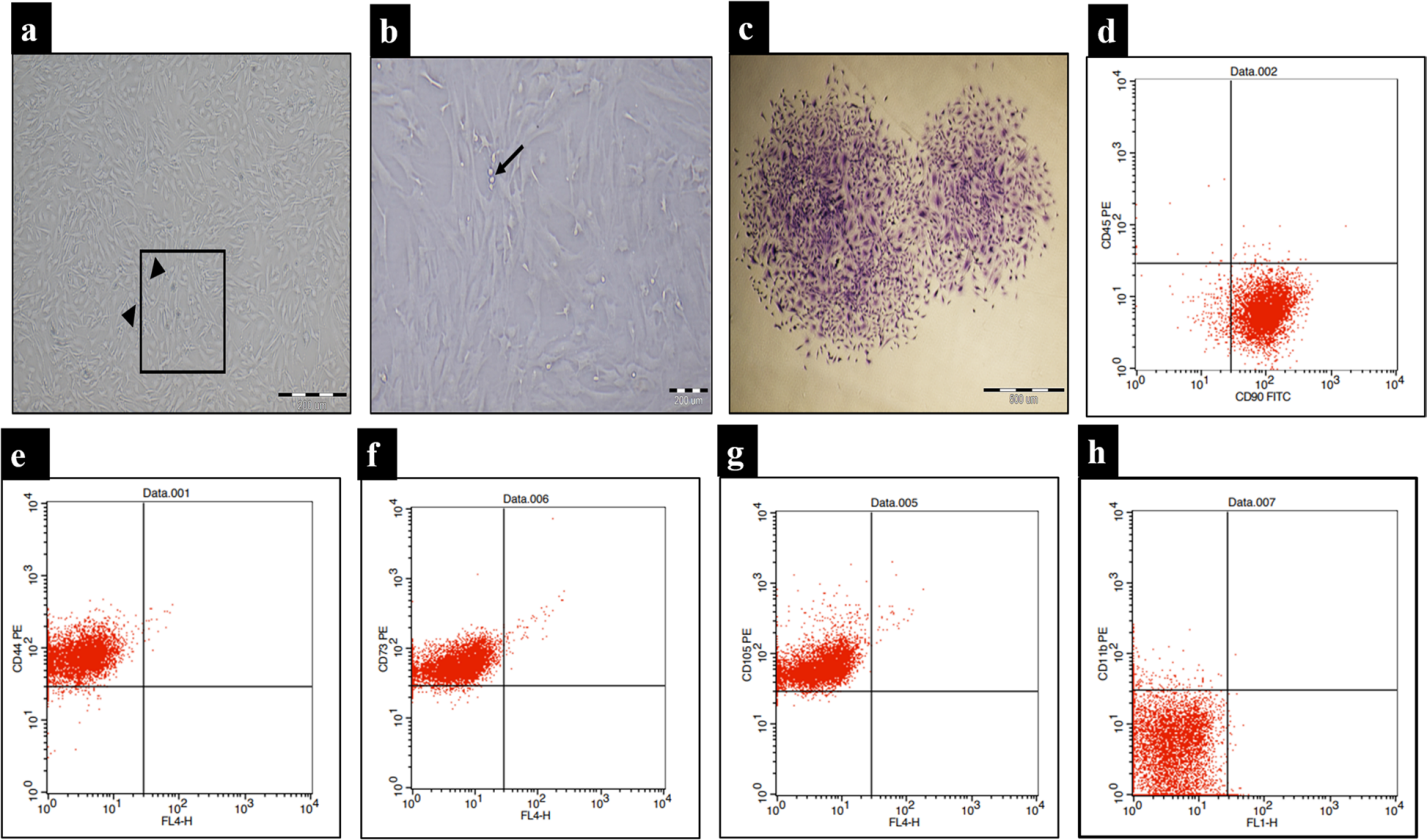
Phenotypic characterization of BMMSC showing spindle-shape, Fibroblast-like cells forming monolayer at P3. MSCs cells 90% confluent (100x), scale bar 200 µm using inverted phase microscope. Arrow head: Proliferating twin cells (**a**), Higher magnification of the previous inset (200x); scale bar 100 µm showing MSCs with fibroblast-like shape. Arrow: Proliferating twin cell (**b**). CFU-F assay, P3 Crystal Violet stain showing two colonies (× 40), scale bar 500 µm (**c**). Representative panel of flow cytometric cell surface markers for BMMSC showing the percentage of CD90, CD45, CD44, CD73, CD105, CD11b cells as 95.03%, 0.01%, 97.92%, 96.17%, 98.90% and 2.4% respectively (**d-h**)

The cell cultures were monitored daily using phase-contrast inverted light microscope. In primary culture, cells were small and rounded, then changed to be spindle in shape after 72 h of culture and reached 70–80% confluence in approximately 10–12 days. With passaging, cell growth was accelerated, and cell morphology showed flattened and spindle-shaped cells. At P3 the culture represented a homogenous fibroblast-like cell monolayer (Fig.1 a,b).

#### Colony forming unit fibroblast assay (CFU-F)

Colony forming unit fibroblast assay for determining cell proliferation and clonogenicity as indicated by a CFU-F potential equals 85% ± 1.93 colonies for BMMSCs after 14 days. (Fig.1c).

#### Immunophenotyping of BM-MSCs

Flow cytometry analysis (FACs caliber, BD) for bone marrow mesenchymal stem cells at P3 revealed: 95.03%, 97.92%, 96.17% and 98.90% of cultured cells expressed mesenchymal cell surface marker CD90, CD44, CD73 and CD105 respectively. Cells were almost negative for the hematopoietic CD45 and CD 11b surface markers where they revealed (0.28% and 2.44% respectively) which are hematopoietic stem cells markers thus cultured cells were typical MSCs (Fig. 1d-h).

### Characterization of extracellular vesicles

#### Transmission electron microscope

**Fig. 2:**
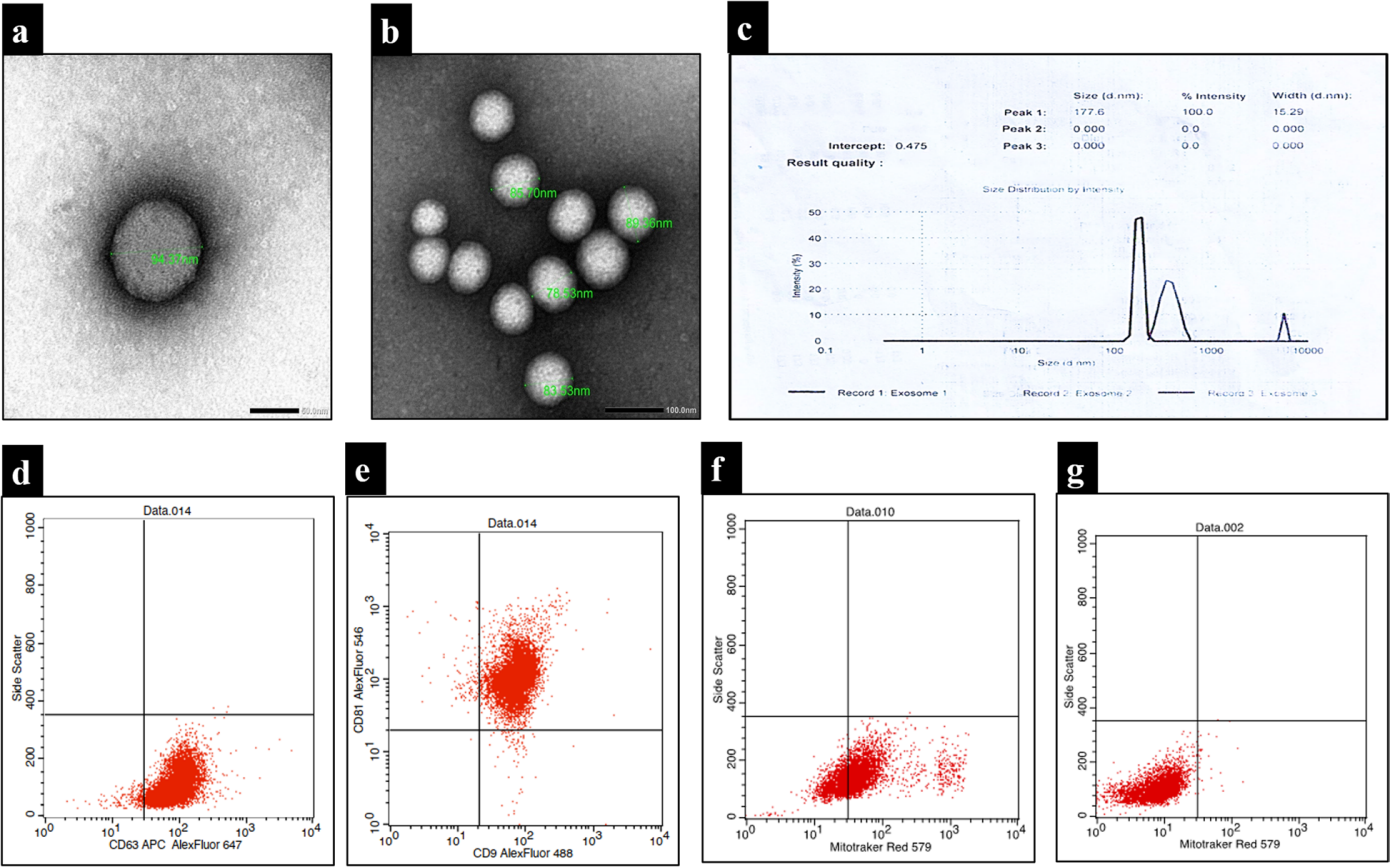
Characterization of extracellular vesicles according to their size and morphology; EVs visualized by TEM showing lipid bilayer EVs (size: 94.37 nm);scale bar 100 nm (**a**) Evs of different sizes ranging from 78–89 nm, Scale bar; 50 nm, (**b**) Intensity analysis curve displaying size distribution and peak size of isolated EVs (**c**). Representative panel of flow cytometric cell surface markers for EVs showing the percentages of CD63 (97.75%) while that of double expressing CD9 and CD81 (97.94%) (**d**,**e**). MitoTracker red-fluorescent dye is used as a negative marker for characterizing the EVs which revealed their presence in the BM-MSCs (+ ve control) (67.11%) while they were detected in the EVs (0.73%) (**f**,**g**)

The presence of extracellular vesicles was confirmed using a transmission electron microscope, which revealed rounded structures of varying sizes with evident lipid bilayer. these represent the appropriate size, shape for extracellular vesicles [42] (Fig. 2a, b).

#### Zeta sizing

Nanoparticles with a peak size of 177.6 nm and an intensity of 100 percent were seen by using zeta sizing. This particle size distribution is typical for EVs (Fig. 2c).

#### Protein content

The protein content of the isolated extracellular vesicles was used to quantify the amount of EVs. Bicinchoninic acid (BCA) assay revealed a protein concentration of 730 g/ml, indicating a high concentration of extracellular vesicles and the efficiency of the isolation procedure.

#### Immunophenotyping of EVs

The expression of tetraspanins on exosomes demonstrated that the percentages of CD63 was 97.75% while that of double expressing CD9 and CD81 was 97.94% (Fig. 2d,e). while the negative marker used for characterizing the EVs was done by using a Mito Tracker (life technologies cat# M7512,USA) which is a red-fluorescent dye that stains mitochondria which revealed their presence in the BM-MSCs (+ ve control) (67.11%) while they were detected in the EVs (0.73%) (Fig. 2f,g).

### Histological results

#### Control group (CG)

**Fig. 3:**
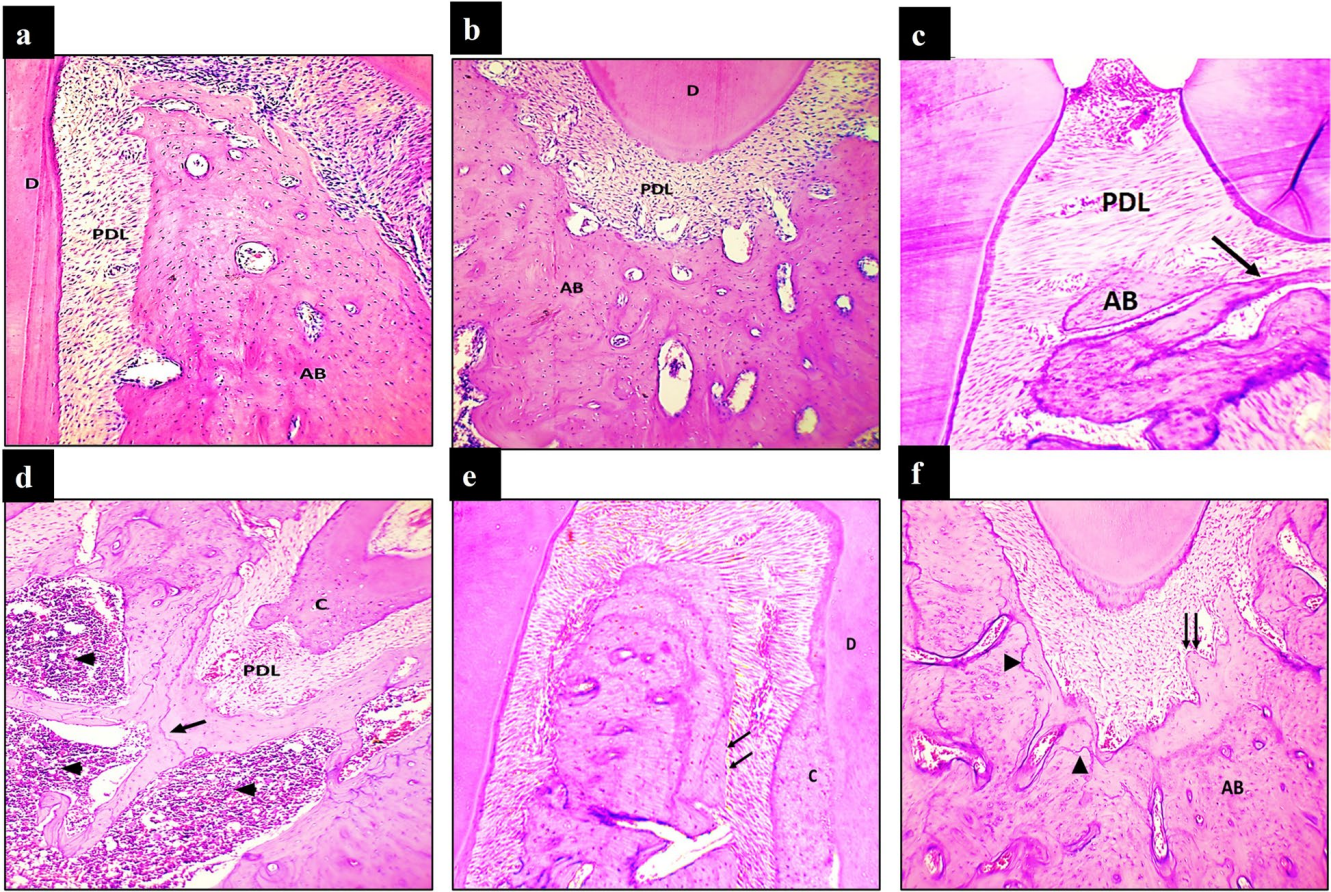
Light microscopic examination. Control group shows smooth boundary at the coronal (**a**) and apical parts (**b**) of the alveolar bone facing the periodontal ligament. Osteocytes are normally distributed. (H&E × 100) The alveolar crest in GOG shows apical displacement of the alveolar bone and irregularity of the alveolar bone surface and thinning of the bony trabeculae (black arrow) (**c)** with the apical region showing irregular bone surface with marked bone resorption. Wide bone marrow spaces with thin bone trabeculae are seen. Note the deeply stained incremental lines (white arrows). Inflammatory cell infiltrate is also evident (arrowheads) (**d**). (H&E × 100) The alveolar crest and middle region of the alveolar bone in ExTG show regular and smooth outline of the alveolar bone. The osteoblastic layer exhibits a regular appearance. (H&E × 100) (**e**). The apical region of the alveolar bone shows normal architecture of the bone surface lined by a continuous layer of osteoblast cells (arrows). Note the presence of incremental lines (white arrows). (H&E × 100) (**f**). AB: Alveolar bone, PDL: Periodontal ligament

Results obtained from the control specimens showed normal alveolar bone structure from the alveolar crest coronally to the apical partof the alveolar bone. The alveolar bone appeared normal with a continuous layer of active osteoblasts lining the bone surface. Osteocytes were regularly distributed with normal sized lacunae. The cancellous bone showed thick regular bony trabeculae enclosing normal, cellular and highly vascularized bone marrow spaces lined by flat endosteal cells (Fig. 3a,b).

#### Glucocorticoid-induced osteoporosis group (GOG)

Histological examination of this group from the cervical margin of the alveolar bone till the apical region showed irregular outline indicating bone resorption and shifting of the crest apically. The bone trabeculae were thin and revealed deeply stained reversal lines.

Osteoblastic cell layer showed a discontinuity along the surface of the alveolar bone in comparison to the control group. Osteocytes were seen with empty or enlarged lacunae containing pyknotic nuclei. Moreover, Osteoclast cells were seen in some regions along the borders occupying the How ship’s lacunae. Cancellous bony trabeculae were irregular and thin enclosing less vascular bone marrow spaces infiltrated with inflammatory cells (Fig. 3c,d).

#### Extracellualr vesicles treated group (ExTG)

Light microscopic findings of extracellular vesicles group showed a generalized restoration of the normal structural features of the alveolar bone. The alveolar bone restored its normal and regular outline from the alveolar crest till the apicalregion of the alveolar bone. The osteoblastic layer restored its continuity along the bone surface. Incremental lines were also evident. Normal osteocytic density and distribution was also seen. The interdental bone exhibited smooth and regular surface on one side while the other side showed some irregularities. The interradicular bone also showed a well-organized bone trabecula. The cancellous bone restored its regular and normal trabeculation enclosing the bone marrow spaces. Normal cellularity and vascularity of the bone marrow was also evident (Fig. 3e,f).

### Scanning electron micrograph results

#### Optimizing group

**Fig. 4:**
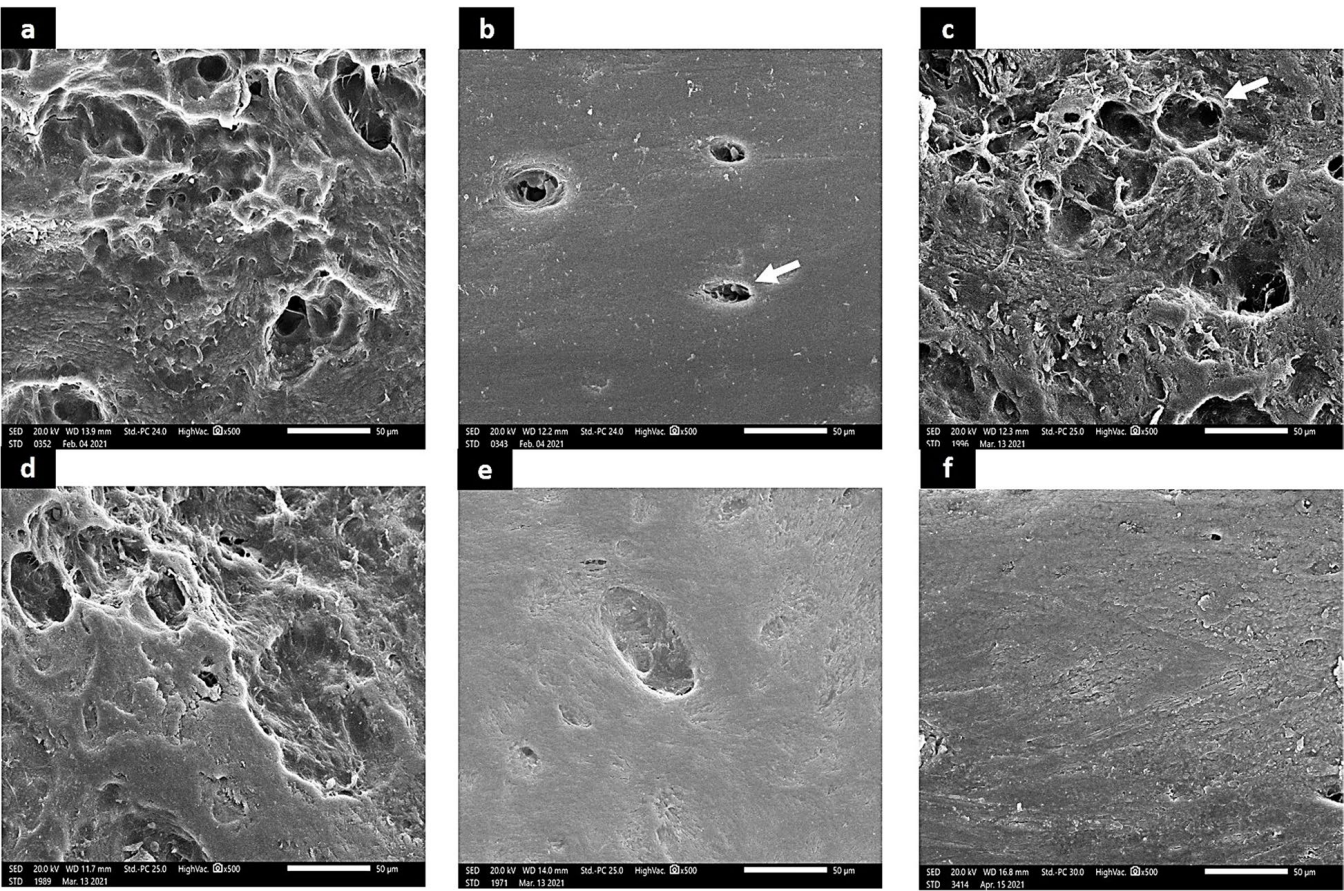
Scanning electron micrograph. Optimizing group showing rough, irregular and destruction in the buccal cortical plate (**a**). The buccal cortical plate in the control group shows a regular and smooth bone surface topography uniform outline of the nutritive canal (arrowhead) (× 500) (**b**). Generalized roughness and porosity is noted in the buccal cortical plate in GOG group with irregular border of the nutritive canals (arrowhead). Massive abrasion and discontinuation of the bone surface is also observed (× 500) (**c**,**d**). the buccal cortical plate in the ExTG shows enhanced bone surface topography and restoration of the normal architecture. Decrease in the surface roughness is noted. Slight irregularities at the internal border of the nutritive canals (× 500) (**e**,**f**)

The optimizing group showed rough, irregular outline of the buccal cortical plate with multiple resorptive regions indicating loss of the normal bone architecture (Fig. 4a).

#### Control group (CG)

The alveolar bone buccal cortical plate surfaces revealed a generalized pattern of smooth and normal surface topography with a regular and well-defined nutritive canal (Fig. 4b).

#### Glucocorticoid-induced osteoporosis group (GOG)

The buccal cortical plates showed generalized roughness and porosity. Irregularly outlined nutritive canals was evident. Areas of smooth bone surface were associated with rough surfaces indicating bone resorption. Multiple resorptive pits with irregular resorptive craters, porosities and deep areas of erosions were seen covering the buccal cortical plate. The buccal cortical plate showed loss of the normal bone architecture (Fig. 4c,d).

#### Extracellular vesicles treated group (ExTG)

The buccal cortical plate showed restoration of the normal architecture. Partial restoration of the bone surface topography was observed. Decrease in the surface roughness was noted with increase in the smooth areas along the bone surface. Slight irregularities were seen in certain areas and at the internal border of the nutritive canals. Some homogenous and smooth bone surface areas free of defects were noticed alternating with roughened irregular areas that exhibited shallow depressions (Fig. 4e,f).

### Energy dispersive x-ray microanalysis

**Fig. 5:**
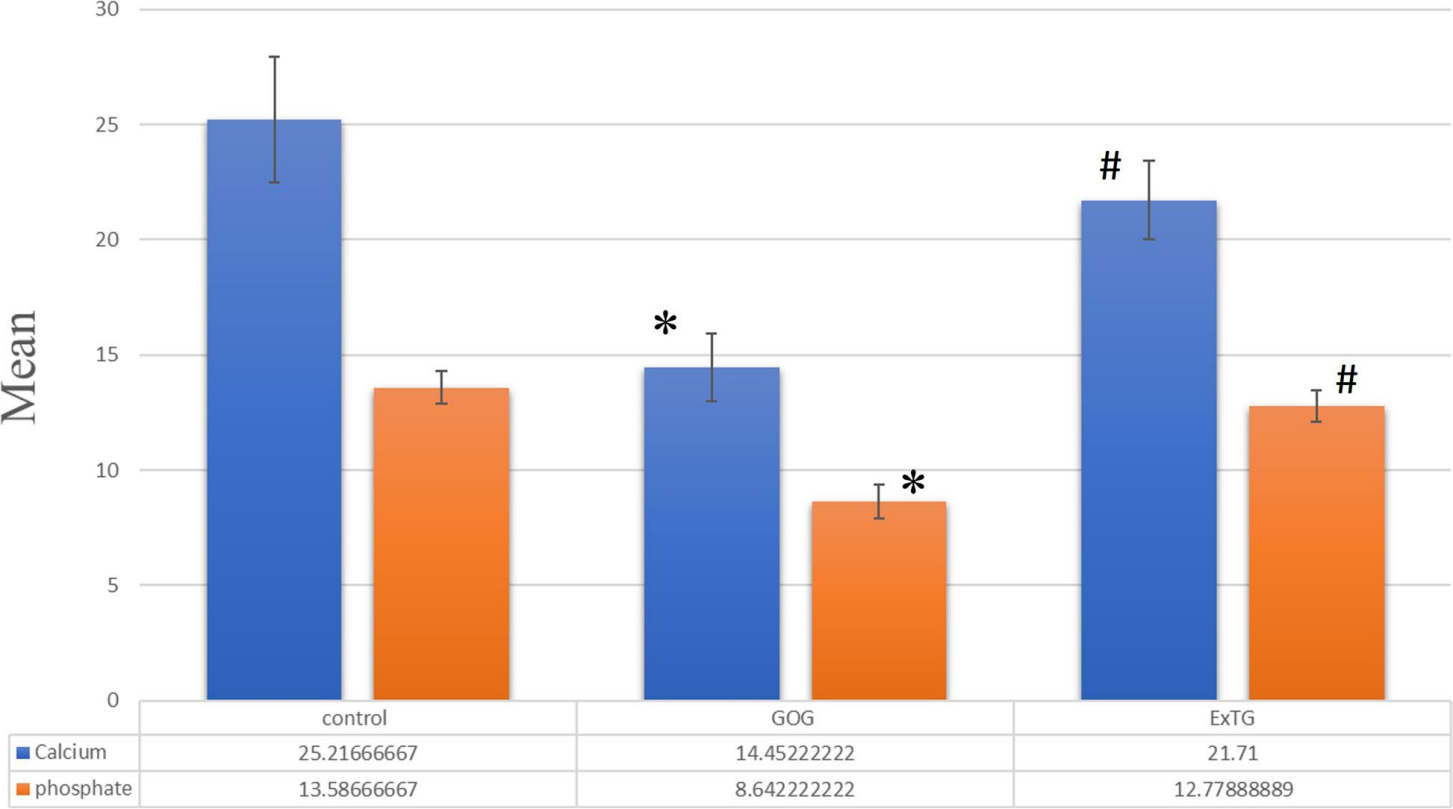
Energy dispersive x-ray microanalysis represented in the bar chart showing Calcium and Phosphate levels, * significantly different from control (CG), # significantly different from GOG, *p* < 0.001

The energy dispersive x-ray microanalysis revealed differences in the surface elemental composition in the different groups. The CG, GOG and ExTG groups showed mean ± standard deviation of calcium level of 25.22 ± 2.73, 14.45 ± 1.45, 22.97 ± 1.54 and mean ± standard deviation of phosphate level of 13.59 ± 0.70, 8.64 ± 0.74, 12.78 ± 0.69 respectively. When compared to CG, the GOG had a statistically significant lower calcium and higher phosphate levels (*p* < 0.001). On the other hand, there was no statistically significant difference between ExTG and CG for calcium and phosphate levels *p* = 0.079 and p = 0.103 respectively.On the contrary, Calcium level was significantly higher and phosphate level was significantly lower when comparing ExTG to GOG (*p* < 0.001) ( Fig. 5).

## Discussion

Extracellular vesicles are nanoscale, membrane bounded vesicles. Exosomes were previously thought to be cellular waste or cell damage byproducts with no effect on neighbor cells. These EVs were recently discovered to be functional vehicles carrying a complex set of proteins important in cellular processes [49].

Extracellular vesicles have variable size (30 to 150 nm) in diameter. They are characterized by their unique content of lipids, proteins, micro-RNA, DNA, and enzymes. The molecular content of extracellular vesicles varies according to the cell of origin.

Extracellular vesicles are signaling-correlated organelles that are released by many cell types. All organisms produce extracellular vesicles and both normal and dying cells release membrane-bound vesicles such as exosomes, micro vesicles, and apoptotic bodies. EVs are characterized by their size (40–100 nm for exosomes, 100–500 nm diameter for the larger micro vesicles, and 500 nm–2 µm for apoptotic bodies) [50]. EVs have multiple functions as playing a role in immune response, wound healing, can be used as a diagnostic tool and as a drug-delivery system [51].

However, The literature has few research on the effect of exosomes on alveolar bone regeneration.

Therefore, the present study investigated the effect of extracellular vesicles derived from BMMSCs on the structure of alveolar bone in rats with induced calcium deficiency. This was done by using histological examination, scanning electron microscopy and elemental microanalysis.

The animal model chosen in this experiment was rats due to their anatomical, physiological, and genetic similarities to humans. They are also chosen because they are readily available, inexpensive, have a relatively long life span, and have a well-defined skeleton [52]. According to Gomes et al. (2011) [53], the reduced lifespan of rodents allows for the study of ageing in bone metabolism and regeneration processes. Also, they stated that rodents are used for in vivo testing of regenerative and/or therapeutic approaches to the bone tissue. Nonetheless, there are some differences between humans and rats. In rats, the mandible is elongated with low bone height. The ramus has minimal thickness with significant muscular attachment [54].The basal bone and the alveolar process are significantly defined in humans unlike rats. The medullary spaces are abundant in case of humans. Also, the bone marrow spaces has more hematopoietic cells than in rats. Moreover, bone regeneration in rats occurs at a higher rate than humans [55].

In this study, glucocorticoid-induced osteoporosis was done by using methyl prednisolone acetate. Concerning the dose of the drug used in this study, it was 40 mg/kg intramuscularly in the quadriceps muscle, three times per week for 3 weeks. This dose was given based on previous studies made by Motomura et al. (2008) [56] and Liu X et al. (2017) [57] who used the same dose to estimate the effect of methylprednisolone on the femur in rabbits and rats respectively.

The optimizing group was assigned to assess the efficacy of the dose and the duration of the drug that was going to be used in the present experiment. The specimens were evaluated by using scanning electron microscope where the buccal cortical plate showed signs of bone destruction and resorption that was represented as irregular and rough buccal cortical plate with multiple resorptive pits and craters. These results indicated that the dose and duration of glucocorticoids used can elicit negative effects on the structure of alveolar bone.

Moreover, there are several methods for the isolation of extracellular vesicles; however, extracellular vesicles were isolated successfully, and the large apoptotic bodies were removed using differential ultra-centrifugation. Transmission electron microscopy was used to characterize the EVs, which had a round morphology with a lipid bilayer and a nano size ranging from 78 to 95 nm. This is in accordance with Van Der Pol et al. (2010) [58] who investigated optical and non-optical methods for detecting and characterizing microparticles and exosomes; they concluded that EVs can be viewed by transmission electron micrograph due to its high resolution capacity which can detect nanoparticles; therefore, it’s an important mean in viewing the morphology and size of exosomes.

Furthermore, the protein content of EVs as measured by the BCA assay was 730 g/ml, indicating the rich protein content of extracellular vesicles and efficiency of the isolation procedure. Stillwell et al. (2013) [59] confirmed the results of the current study as they studied biological membranes and concluded that protein accounts for 25–75 percent of the weight of various biological membranes, with most membranes containing approximately 50 percent protein. Also, Smyth et al. (2014) [60] demonstrated that proteins have a role in adherence and internalizing EVs by the recipient cell. Moreover, Zeta sizing revealed nanoparticles with a peak size of 177.6 and intensity of 100%. The results of the present study are in accordance with Khodashenas et al. (2019) [61] who studied different characterization methods for the detection of EVs for diagnostic and therapeutic applications.

Histological results of this study revealed that the control group had a regular and normal alveolar bone architecture with active plump osteoblasts lining the bone surface. Osteocytes were regularly distributed. Incremental lines were also evident. It also showed normal trabeculations of the cancellous bone with highly cellular and well vascularized bone marrow spaces This is in line with Capulli et al. (2014) [62] who illustrated the mechanisms regulating osteoblasts and osteocytes functions.

On the other hand, the glucocorticoid-induced osteoporosis group showed bone resorption and destruction of the normal bone architecture. The osteoblasts showed loss of continuity along the bone surface. This is in line with Briot et al. (2015) [63] who analyzed the underlying mechanisms of glucocorticoids-induced osteoporosis and illustrated that glucocorticoids induce apoptotic effects in osteocytes and osteoblasts by activating caspase 3. Additionally, Yang et al. (2018) [64] investigated the molecular mechanisms underlying GC-induced modulation of osteogenesis in female rats and they concluded that in the presence of GCs, bone marrow stromal cells are directed towards the adipogenic rather than the osteoblastic lineage, which is due to the induction of peroxisome proliferator-activated receptor 2 (PPAR 2), which have an important role in adipogenesis. Chotiyarnwong et al. (2020) [65] elaborated the pathogenesis of glucocorticoids-induced osteoporosis and they found that GCs decrease osteoblasts count and impair its function which led to a decrease in bone formation. They also added that GCs impair osteoblasts differentiation and maturation.

The current study also revealed pyknotic nuclei in some osteocytes with some lacunae appearing empty. The findings of the present study are consonance with Weinstein et al. (2012) [66] who hypothesized that GCs cause osteocyte loss, which disrupts the canalicular network in osteocyte and results in a failure to detect signals related to bone remodeling. In addition, they claimed that glucocorticoids cause apoptosis in osteocytes.

Nevertheless, the histological results of the ExTG group showed that the alveolar bone restored its regular, smooth outline to a great extend close to the control group, with deeply stained incremental lines indicating bone remodeling. The bone trabeculae were well developed surrounding cellular and vascular bone marrow spaces.

Moreover, Furuta et al. (2016) [67] confirmed the results of the current study as they studied the effect of EVs on fracture healing in a mouse model and they presumed thatEVs promote angiogenesis by increasing the expression of VEGF and HIF-1α and thereby, they promote osteogenesis in the defect site.

Furthermore, Takeuchi et al. (2019) [68] studied the effect of exosomes derived from BM mesenchymal stem cells on osteogenesis and angiogenesis in a rat model with clavicular defect and hypothesized that EVs promote the formation of bone by increasing the expression of osteocalcin and Runx2. Also, they found that exosomes increase ALP activity and mineral deposition.

In addition, another study was done by Zhang et al. (2020) [69] on the healing potential of EVs in non-union fracture in rat model, and the found that EVs promoted regeneration of bone by enhancing cell survival, migration and differentiation. They also increase the expression of osteocalcin and osteopontin.

Scanning electron microscope (SEM) results supported the histological findings as it showed that the control group revealed a smooth and regular bone surface topography. These results are confirmed by Zhou et al. (2018) [70] who studied the normal morphological features of alveolar bone in rats.

On the other hand, the glucocorticoid- induced osteoporosis group showed an irregular surface with resorptive pits and craters in the outer cortical plate indicating massive bone resorption associated with GCs. These results are in accordance with Yoon et al. (2012) [71] who studied the structure of bone in mice under GCs treatment and they concluded that GCs lead to decrease the formation of bone and accelerate resorption of bone.

However, SEM of the ExTG group showed an improvement in bone surface topography with minor irregularities in the outer cortical plate. These results are confirmed by Yuan et al. (2021) [72] who studied the effect of hypoxia-preconditioned mesenchymal stem cells on steroid-induced osteonecrosis in the femoral head of rats and they concluded that EVs promote angiogenesis and osteogenesis through increased expression of growth factors and the high protein cargo in theEVs.

The results of the elemental microanalysis match the results of the scanning electron microscope. According to Scimeca et al. (2018) [73] energy dispersive x-ray microanalysis is an effective method for determining the spatial distribution and abundance ratios of calcium (Ca) and phosphorus (P). In the glucocorticoid- induced osteoporosis group, the EDX showed decrease in calcium concentration when compared to phosphorous. Nevertheless, the EVs group showed an increase in calcium levels and a relative restoration of the normal calcium-phosphorous ratios in comparison with the glucocorticoid- induced osteoporosis group. 

## Conclusion

Extracellular vesicles derived from bone marrow mesenchymal stem cells provide a promising role in alveolar bone regeneration where it can restore the normal mineral balance in the alveolar bone and they have a positive effect in treating glucocorticoid-induced osteoporosis by preventing alveolar bone resorption and restoring its normal architecture however the clinical relevance of these findings are not known and should be acknowledged.

## Data Availability

The data used and/or analyzed during the current study are contained within the manuscript.

## Abbreviations

EVs: Extracellular vesicles
EDX: Energy dispersive x-ray
M-CSF: Macrophage colony-stimulating factor
GCs: Glucocorticoids
BMMSCs: Bone marrow-mesenchymal stem cells
PTH: Parathyroid hormone
GFs: Growth factors
TGF-β: Transforming growth factor beta
BMPs: Bone morphogenetic protein
FGF-2: Fibroblast growth factor-2
